# Cancer resistance to treatment and antiresistance tools offered by multimodal multifunctional nanoparticles

**DOI:** 10.1186/s12645-017-0030-4

**Published:** 2017-10-26

**Authors:** Eudald Casals, Muriel F. Gusta, Macarena Cobaleda-Siles, Ana Garcia-Sanz, Victor F. Puntes

**Affiliations:** 1 Vall d’Hebron Research Institute (VHIR), Passeig Vall d’Hebron 119-129, 08035 Barcelona, Spain; 2 Catalan Institute of Nanoscience and Nanotechnology (ICN2), CSIC and BIST, Campus UAB, Bellaterra, 08193 Barcelona, Spain; 3 Catalan Institution for Research and Advanced Studies (ICREA), Barcelona, Spain

**Keywords:** Cancer resistance, Resistance to treatment, Inorganic nanoparticles, Multifunctional, Multimodality

## Abstract

Chemotherapeutic agents have limited efficacy and resistance to them limits today and will limit tomorrow our capabilities of cure. Resistance to treatment with anticancer drugs results from a variety of factors including individual variations in patients and somatic cell genetic differences in tumours. In front of this, multimodality has appeared as a promising strategy to overcome resistance. In this context, the use of nanoparticle-based platforms enables many possibilities to address cancer resistance mechanisms. Nanoparticles can act as carriers and substrates for different ligands and biologically active molecules, antennas for imaging, thermal and radiotherapy and, at the same time, they can be effectors by themselves. This enables their use in multimodal therapies to overcome the wall of resistance where conventional medicine crash as ageing of the population advance. In this work, we review the cancer resistance mechanisms and the advantages of inorganic nanomaterials to enable multimodality against them. In addition, we comment on the need of a profound understanding of what happens to the nanoparticle-based platforms in the biological environment for those possibilities to become a reality.

## Introduction

Cancer is one of the leading causes of morbidity and mortality worldwide and it is expected to become the major cause of death in the coming decades (NIH 2017; WHO 2017). Cancer is defined as a multifactorial disease involving a malignant growth of tissue (malignant tumour) that possesses no physiological function, and arises from an uncontrolled, usually fast, cellular proliferation. The tumour can expand locally in the same tissue by cellular invasion and systemically to other organs, a process known as metastasis. In cancer, the cellular mechanisms that regulate gene expression and cell proliferation are altered, mostly due to mutations of the genetic material or other epigenetic modifications. The cell type and these alterations are what will mainly determine tumour’s growth rate and metastatic potential, and consequently severity. However, other factors such as the patient hormone profile or immune system characteristics can be determinant in the individual clinical development of cancer, increasing its intricacy and pledging for personalized treatments (Greaves 2000).

Besides, the considerable progress made in understanding the biological and molecular basis of cancer during the past 50 years has not been translated into a notable improvement of its incidence and mortality (Kiberstis and Travis 2006), neither in the control of treatment-limiting side effects, also contributing to improper treatment compliance (Frenkel 2013). Therefore, efficient cancer therapies still remain elusive. Ideally, cancer treatments aim to entirely eliminate all tumour cells, minimizing side effects on the rest of the organism. Surgery, radiotherapy, and chemotherapy have been the main treatment approaches used in the past decades. Today, along with them, other forms of therapy as hormone therapy, immunotherapy, photodynamic therapy and targeted therapies complete the catalogue of treatment modalities used in the clinic to fight cancer.

## Cancer resistance mechanisms

The main obstacle for the success of cancer treatment is the development of resistance through different mechanisms. Briefly, drug resistance is the reduction in effectiveness of a drug such as an antimicrobial or an antineoplastic (antitumoural) agent in curing a disease or condition. Based on the initial tumour response to a treatment, mechanisms of cancer resistance can be classified in two categories: (a) *intrinsic*, which is the resistance due to features present in the tumour before the therapy. Consequently, in this case, the tumour will be resistant even before being treated. (b) *Acquired*, which is the resistance developed as a response to the selective pressure of the treatment. In this case, usually tumour size is initially reduced as the *bulk* of the tumour is eliminated. However, some clones evolve and develop resistance, remain latent until the treatment is finished, and then expand to repopulate the tumour (Livney and Assaraf 2013; Thomas and Coley 2003). Also, it may happen a combination of both. There are some tumour subpopulations that show intrinsic resistance, but as treatment starts it acts as an agent exerting selective pressure, and some other populations will acquire resistance de novo. In other words, attacking the tumour may train it and make it more robust and resistant, as happens with bacteria if the antibiotic treatment is interrupted before completeness (Liang et al. 2010).

Disease resilience is a result of genetic diversity. In recent years researchers learned that within a single tumour or infection, there is great genetic diversity and variation among clusters of cells. This recalls the Darwinian laws of natural selection, the survival of the fittest. Diseases related to or produced by life forms, such as cancer, are subjected to them. Thus, as medical practice for cancer treatment increases and improves, resistance rises, in an *arms race* vicious loop.

Recent studies revealed tumour heterogeneity as an important driver for the onset of resistance (Hanahan and Weinberg 2011; Nagy and Dvorak 2012; Saunders et al. 2012; Yachida et al. 2010; Zhu et al. 2014), which can make the whole tumour as resistant to treatment once the first wave of treatment has eliminated the weak tumoural cells, leaving the resistant ones alone. The tumour heterogeneity is the observation of the high genetic variation, which is translated into distinct morphological and phenotypic profiles, different cell plasticity, metabolism, motility, proliferation activity, and metastatic potential of the different cells that constitute the whole tumour. The tumour heterogeneity is critically determined by the microenvironment in which the tumoural cells reside (Bissell et al. 2002; Joyce and Fearon 2015; Levental et al. 2009; Spill et al. 2016; Wiseman and Werb 2002). It is in all this fronts that nanotechnology needs to provide ways of action.

There are different molecular mechanisms and adaptive responses involved in the onset of resistance. Some of these processes are skilful cellular mechanisms that make the *tumoural cell resistant*. Additionally, due to the diversity of genetic populations and the microenvironment in a tumour, in other cases it is the *tumour tissue that becomse resistant*. Several mechanisms that make tumoural cells resistant to chemotherapeutic treatments have been already identified, such as increased rates of drug efflux, altered drug metabolism and target, and repairing mechanisms. In the case of increased drug efflux, the overexpression of transporters in the cytoplasmic membrane expels the chemotherapeutic agents from the tumoural cell before they can act. The most paradigmatic and known case is the role of the P-glycoprotein (P-gp), a cell membrane protein that acts as an ATP-dependent drug efflux pump, decreasing drug accumulation. The P-gp mechanism of action was first described in the work of Juliano and Ling (1976) using Chinese hamster ovary cells selected for resistance to colchicine. They found that this drug-resistant phenotype displayed a membrane alteration that reduced rates of drug permeation, and that the relative amount of P-gp correlated with the degree of drug resistance in a number of independent mutant clones. Futher, many other works proved the role of P-gp in the appearence of drug resistance (Doyle et al. 1998; Robey et al. 2007, 2008).Other mechanisms involve alterations in drug metabolism, thus reducing drug activity (Kato et al. 1963; Pao et al. 2005b; Toffoli et al. 2010), the mutation and alteration of drug targets (Greenman et al. 2007; Pao et al. 2005a), and the activation and up-regulation of alternative compensatory signalling pathways (Fojo and Bates 2003; Gottesman et al. 2002; Longley and Johnston 2005). For instance, the up-regulation of oncogenes and the higher DNA repair capacity have been proved to make some tumoural cells more resistant to drugs promoting apoptosis (Cantley and Neel 1999; Evan and Littlewood 1998; Harris 1996; Zhao et al. 2004).

Many of these mechanisms are not drug-specific and cancer cells are constantly using a variety of non-specific tools, involving genes, proteins, and altered pathways, to ensure their survival against antineoplastic drugs and treatments. Thus, usually, tumoural cells are resistant to drugs of a wide chemical variety, what is known as multidrug resistance (MDR) from where over 90% of cancer treatment failures have been attributed to (Gong et al. 2012; Luqmani 2005). Note that the list of mechanisms mentioned above that induce the appearance of drug resistance and MDR is not exhaustive and new studies of drug resistance mechanisms are constantly appearing, as well as the complex and challenging ways in overcoming this type of multidrug treatment resistance (Bachas et al. 2017; Dlugosz and Janecka 2016; Gao et al. 2015a, b; Higgins 2007; Noll et al. 2017).

In this regard, recently, cancer stem cells (CSC) (alternatively named “tumour-initiating cells”) have been identified as another source of tumour tissue resistance (Beck and Blanpain 2013; Greaves and Maley 2012; Hanahan and Weinberg 2011). CSC were first described in the work of Bonnet and Dick (1997) where they showed a cellular population capable of initiating human acute myeloid leukaemia in non-obese diabetic mice with severe combined immunodeficiency disease. These cells were showed to possess the potential for self-renewal and differentiative and proliferative capacities expected for leukaemic stem cells. CSC represent a small population of cancer cells and share common properties with normal stem cells. CSC are usually quiescent (as normal stem cells) and provided with different characteristics that makes them intrinsically multi-drug resistant. Mechanisms of action of most of chemotherapeutic agents rely on dividing cells in order to cause lethal damage and induce apoptosis by altering the cell cycle. Thus, CSC are less susceptible to therapies. In addition, described different signalling pathways contributing to maintain the stemness of CSC, guaranteeing chemotherapy resistance, tumour growth, and metastases (Shipitsin and Polyak 2008; Valent et al. 2012; Vinogradov and Wei 2012) have been described. Therefore, CSC have been considered intrinsically drug resistant, and once the treatment is stopped they have the ability to regenerate the tumoural tissue again and again.

Furthermore, the local tumour microenvironment has been shown to decisively contribute to cancer growth, metastasis, and progression to resistance (Bissell et al. 2002; Wiseman and Werb 2002). It constitutes the cellular environment in which the tumour exists that includes among others the surrounding blood vessels, stromal cells such as the fibroblasts, immune cells, and the extracellular matrix and signalling molecules present in it. As it happens with normal tissues and the extracellular matrix, the tumour and the surrounding microenvironment are closely related and interact constantly. Tumoural cells influence the microenvironment by releasing extracellular signals, promoting tumour angiogenesis and inducing peripheral immune tolerance. In turn, the immune cells in the microenvironment can affect the growth and evolution of tumoural cells. Thus, through this interaction, the production of secreted factors by tumoural cells can increase the capacity of the microenvironment to alter the response of the tumour to treatment (Iyer et al. 2013; Swartz et al. 2012; Vinogradov and Wei 2012). This surrounding tumour area is an appealing target for nanoparticles (NPs) that reach the tumour periphery by enhanced extravasation, penetration, and retention effect (Maeda 2001). Table 1 shows a summary of the adaptation and resistance mechanisms described in this work.

**Table 1:** Description of the main mechanisms of cancer resistance, and treatment approaches offered by multifunctional nanoparticles

Resistance mechanism	Description	NP-based treatment approach
Drug metabolism and drug target regulation	Anti-cancer treatments can induce the up-regulation of certain signalling pathways in order to develop resistance like amplification, drug metabolism or mutation of drug targets (Vinogradov and Wei 2012)	*Drug protection and drug cocktail* Drugs can be conjugated with NP for drug delivery, which protects them from degradation. Further, when combining more than one drug in a single NP lowers the chances of resistance onset
Efflux pumps	Drug efflux transporters—first described in reference (Juliano and Ling 1976)—in the cytoplasmic membrane that expel the chemotherapeutic agents from the cell are generally found to be overexpressed in MDR cancer cells, lowering intracellular drug concentration (Kirtane et al. 2013)	*Drug cocktail and drug cargo* The local release of (different) drugs from the NP increase intracellular drug concentration, which can saturate the efflux pumps minimizing their resistance effect
Tumour microenvironment	The cellular environment in which the tumour exists can alter the response of tumour cells to chemotherapy and targeted therapies. It induces the production of secreted factors, which drives tumour growth, MDR and metastasis. Also, it creates a suitable environment for treatment resistance due to the high interstitial pressure (impeding drug penetration) and hypoxia (up-regulating other resistance signalling pathways) (Iyer et al. 2013; Swartz et al. 2012; Vinogradov and Wei 2012)	*Improved tumour penetration* NPs enable local hyperthermia which can contribute in (a) increasing the blood flow and tumour oxygenation, and (b) enhancing drug penetration by decreasing tumour viscosity
Quiescent phenotypes	As conventional chemotherapy agents rely on blocking cell division to induce apoptosis, quiescent cells are not affected. Once the treatment is stopped, this remaining pool of cells can grow to repopulate the tumour. A significant tumour subpopulation displaying this phenotype are CSC, which also possess other intrinsic resistant properties (Dean et al. 2005)	*Radio-enhancement* Radiotherapy and hyperthermia are efficient cancer treatments irrespective of cellular type. NPs act as sensitizers in thermo and radio, increasing the local damage to kill more resistant cells
Stemness	There are several signalling pathways and genes involved in CSC maintenance. The most studied are Hedgehog, Wnt, Notch, and Nanog. Different studies showed that they provide the necessary signals to remain in the stem cell state to self-renew, to guarantee tumour growth and they have also been related to chemotherapy resistance and metastases (Vinogradov and Wei 2012)	*Targeting. Side effects attenuation* (1) NPs can enable targeted therapies, which increases local damage at the targeted site while attenuating side effects in the rest of the organism. This reduces side effects and allows to fight highly resistant cells by (a) increasing doses of drugs and/or radiation (more aggressive treatment) and, (b) combining effects of chemotherapy (drug cocktails), gene therapy, and radiotherapy (overwhelming resistance and repairing mechanisms) (2) Although these are not approaches to address these specific resistance mechanisms, is the NP which enables multimodal treatments able to fight against them
Apoptosis resistance	The up-regulation of oncogenes and the higher DNA repair capacity have been proved to make some tumoural cells more resistant to apoptosis. Additionally, the hypoxic microenvironment has been found to further induce apoptosis resistance, the hypoxia-inducible factors (HIF) up-regulate the factors of DNA-repair machinery (Milane et al. 2011)	

## Multimodality

In this scenario, it is accepted that none of the existing single-modality treatments may cure cancer. Current anticancer therapies (including chemotherapy, radiotherapy, surgery, hormone therapy, immunotherapy, photodynamic therapy, and targeted therapies) are not effective yet in the treatment of tumour resistance (Mi et al. 2012a). Even more, as it has been mentioned above, chemotherapy, alone or even in combination with other therapies, fails to eradicate CSC clones and instead favour the expansion of the CSC pool or select for the MDR resistant cell clones, which ultimately leads to relapse with new tumours becoming more malignant (Clappier et al. 2011). Also, radiotherapy and chemotherapy may be inefficient when tumour cells are not highly susceptible to them due to radio-insensitivity and the MDR intrinsic or acquired by cancer cells (Seiwert et al. 2007). Thus, improved approaches to overcome tumour resistance are increasingly being sought out. To this end, multimodal treatments are being investigated for the possible synergistic effects of the combination of different therapies. Multimodality is understood as the mixture of more than one drug and therapies including thermotherapy, radiotherapy, immunotherapy, and gene therapy. Here, the key is to treat cancer as something that is not uniform and unchanging; therefore, combined attacks (*multimodality*) to the target seem the proper approach (Chiang et al. 2010; Lai et al. 2003). This is because therapeutic effects of the different treatments are designed to add up, while their side effects are distributed. Importantly, there is a reduced probability of resistance generation in the case of multimodality since drug and therapy cocktails target different structures of the tumoural cells and their microenvironment, and have complex action mechanisms to which is more difficult to develop resistance.

As the most common reason for acquisition of resistance to a broad range of different drugs is the expression of one or more mechanisms that detect, deactivate and eject drugs from cells, strategies based on overloading the target with more drugs and therapies are appealing. In addition, side effects avoid this type of approach. In this context, multiple drugs loading onto NPs that protect and transport the drug seem a very promising tool to deliver a *cargo* of drugs to the target cell, overriding defense mechanisms and avoiding side effects. Regarding this last point, while many efforts have been carried out looking for increased efficacy of antitumoural agents, the use of NPs to avoid side effects allowing for extended therapy has been also demonstrated (Comenge et al. 2012). Additionally, the light absorption properties of inorganic NPs can be employed as imaging contrast, radiotherapy*** and thermal therapy agents (Puntes 2016).

### Inorganic NPs as scaffolds

**Fig. 1:**
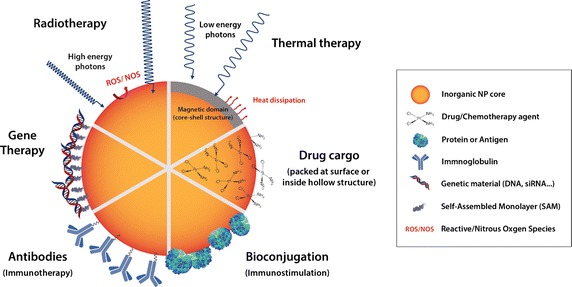
Schematic representation of different cancer treatment modalities that can be integrated in a single NP-based platform

To develop multimodality, inorganic NPs are especially suited to combine in a unique platform different tumour treatment modalities (Fig. 1). Inorganic NPs are small and can, therefore, interact with molecular biological structures in a unique manner (Alivisatos 2001). Thus NP-based platforms can be used as scaffolds where the NP is *at the service of the drug*, to transport and to protect it (Chavany et al. 1994; Han et al. 2006). Not only they are drug cargos, allowing a high dose of drug to arrive at more delayed and intermittent times (Comenge et al. 2012), but also they can modify the biodistribution of the drug in the organs, in the tissues and in the cells, while reducing adverse effects (Comenge et al. 2012). The co-administration and co-delivery of different drugs and biomolecules such as antibodies and genetic materials with NPs not only contribute to an improved accumulation of drugs in the tumour but also unify their pharmacokinetic profiles and limits drug degradation. Also, the transport of the drug with the proper coating of the NP and/or using hollow nanostructures may limit not only systemic degradation of the drugs but also the ejection of drugs from the cells before they act (drug efflux) and other drug-detoxifying mechanisms (Garcia-Fernandez et al. 2017; MacDiarmid et al. 2009; Meng et al. 2010). All these combinatory effects should overwhelm and override the resistance mechanisms of the tumoural cells. For instance, in the work of Meng et al. (2010) they used mesporous silica nanoparticles as a platform to deliver both doxorubicine and siRNA in drug-resistant cancer cell line (KB-V1 cells). As the used siRNA knocks down gene expression of a drug exporter used to improve drug sensitivity to chemotherapeutic agents, this dual delivery was capable of increasing drug concentration intracellularly and in the nucleus to levels exceeding that of free doxorubicin or the drug being delivered in the absence of siRNA. Other examples of these possibilities are in the section of this review “Nanoparticles at work enabling multimodality”.

Indeed it has been postulated that in some cases chemotherapy alone does not kill all tumoural cells, but that the dying tumoural cells are recognized by the immune system which allows their complete eradication (Apetoh et al. 2007). The immune system plays a key role in prevention and elimination of tumours. The immune system can specifically identify and eliminate cancerous or pre-cancerous cells by recognizing mutations or ligands related to stress, a process known as tumour immune surveillance (Smyth 2007). Still, some tumour cells overcome the immune system and expand to develop a whole tumour. Thus, the theory of tumour immune surveillance has recently been updated by emergence of the newer concept of tumour ‘immunoediting’ (Smyth 2007). The process encompasses three steps: (a) elimination corresponds to immunosurveillance; (b) it is followed by an equilibrium phase, where tumour cells with reduced immunogenicity are selected; (c) finally, the escape is the process where the immunologically sculpted tumour expands in an uncontrolled manner in the immunocompetent host (Dunn et al. 2002). The remaining immunogenic cancer cells use different mechanisms to evade immune elimination. For instance, they can secrete TGF-b or other immunosuppressive factors to inhibit cell-mediated immunity of cytotoxic T-lymphocytes (CTLs) and natural killers (NK) (Shields et al. 2010; Yang et al. 2010). Other mechanisms rely on the recruitment of immunomodulatory cells as T-regulatory cells (Tregs) and myeloid-derived suppressor cells (MDSCs) providing an inmuno-protected area where the tumour can keep on growing (Mougiakakos et al. 2010; Ostrand-Rosenberg and Sinha 2009).

The possibilities of the rational control on the functionalization of inorganic NPs with biomolecules is particularly important for cancer immunotherapy, the training of the immune system to attack the tumour (Fan and Moon 2015; Morgan et al. 2006), especially in the case of therapeutic vaccines. Three critical elements are considered to be essential in the composition of an effective vaccine: an antigen to trigger a specific immune response, an adjuvant able to stimulate the innate immunity, and a delivery system to ensure optimal delivery (Reddy et al. 2006). To obtain the full activation of antigen-presenting cells (APCs), the simultaneous action of antigens and adjuvants is critical. In this regard, inorganic NPs can help to develop (a) safe and powerful adjuvants to stimulate the immune system in a non-specific way (Bastus et al. 2009a, b) that induces an inflammatory state able to detect the otherwise evading tumours (Fan and Moon 2015; Jarvinen et al. 2009); and (b) as antigen-presenter platforms (Bachmann et al. 1993), by conjugating them to tumour-associated antigens to develop the adaptive immune response against it (by boosting the immune response through the aggregation and repetition of antigens).

Thus, the possibility of incorporating antigens and adjuvants makes NPs ideal platforms for developing cancer vaccines (Park et al. 2013; Silva et al. 2013). In addition, chemotherapeutic agents can be loaded into them combining immune, chemo, and radio therapy enhancement in a single object. Accordingly, NPs could increase the uptake of antigens by dendritic cells (DCs) which results in enhanced immune responses against tumour. The group of D. Messmer first demonstrated that the conjugation of Hp91 (an already identified immunostimulatory peptide) to poly(d,l-lactic-co-glycolic) acid NPs (PLGA-NPs) significantly enhanced the activation of DCs, compared to free Hp91 (Clawson et al. 2010). More recently, they tested this system against human epidermal growth factor receptor 2 (HER2)-positive breast cancer cells (Campbell et al. 2015). Here, as an effect of higher DCs activation, they observed enhanced activation of HER2-specific (CTL) responses, delayed tumour development, and prolonged survival of the injected mice. Additionally, it has been reported that liposomal NPs can induce a depot effect at the site of injection generating a gradual release of the antigen and, therefore, increasing its exposure to the cells of the immune system (Henriksen-Lacey et al. 2011). This leads to an enhanced APCs recruitment and activation, and also eliminates the need for repeated doses of the vaccine.

Another immune-based cancer therapy approach is the use of antibodies for blocking signalling pathways (Karapetis et al. 2008). In this particular cases, the instability of the exogenous antibodies and their low efficiency calls for nanoconjugation (Bhattacharyya et al. 2010; Garcia-Fernandez et al. 2017). Thus by condensing the antibodies on top of a NP surface, they are protected from systemic degradation (Prego et al. 2010) as their pharmacokinetic profile is altered allowing for improved targeting (Comenge et al. 2012). Additionally, the use of NP-antibody conjugates has shown a prolonged antibody therapy effect by avoiding receptor recycling as well of decreasing the needed antibody dose in the case of Cetuxymab-Au NPs conjugates targeting the epidermal growth factor receptor (EGFR) of A431 cells (Garcia-Fernandez et al. 2017). Coverage density and orientation of antibodies were strictly controlled to properly evaluate their effects. Results showed epithelial growth factor receptor blocking along with their altered trafficking signalling effects. The blocking effects of cetuximab were increased and sustained for a longer time when associated with the Au NPs (Garcia-Fernandez et al. 2017). Here, the use of NP-antibody conjugates also presents some natural advantages: rational design, low toxicity, low-cost, and modified and modifiable biodistribution.

### Inorganic NPs as actuators

In addition, NPs can be active by themselves since they can be antennas that absorb photons of determined wavelengths, to which we are transparent. Thus, radiotherapy effects can be enhanced in such a way that employed doses can be decreased where only the NPs allow the toxic effect, improving localized radiotherapy. In these cases, the coating can be *at the service of the NPs* to transport them to the target site. Inorganic NPs can interact with photons of different wavelengths and trigger a variety of physical processes. Due to the high electronic density of inorganic materials, they can absorb strongly X-rays and selectively enhance the damage inflicted on tumoural tissue in radiotherapy treatments. This is mediated by the fact that these materials absorb strongly the primary radiation beam (typically X-rays in the MeV range, although electrons, neutrons, and positron are also employed), especially high Z number atoms, and subsequently generate a cascade of secondary low-energy electrons (LEEs) highly toxic within a very short range around the NP (Pimblott and LaVerne 2007). These latter are the main source of energy deposition and radiation-induced damage in biological tissue (Sanche 2005). Moreover, even below ~ 15 eV, LEEs can efficiently induce molecular fragmentation into highly reactive free radicals through dissociative electron attachment reactions (Boudaiffa et al. 2000). Within the complex environment of living cells, these light-matter interaction processes can directly affect DNA and other nearby cellular components. Additionally, the irradiated metallic NP can be activated producing catalytically free radicals as hydroxyl radicals OH· and hydrogen peroxide H_2_O_2_ among others (Von Sonntag 2006), which can initiate further reactions and induce oxidative stress and cellular damage (Boudaiffa et al. 2000; Von Sonntag 2006). Thus, heavy atom irradiated NPs can be seen as a source of free reactive radicals, as pointed out by Carter et al. (2007) and also as a vehicle of direct damage as proposed by Sanche group (Brun et al. 2009).

Recent works studying the effects of Au NPs in combination with radiation in various cell lines found a damage enhancement factor between 1.5 and 3.4 times depending on the size of the NPs (Chithrani et al. 2010), incident energy (Rahman et al. 2014), and cell type (Bobyk et al. 2013; Hainfeld et al. 2008). Further studies performed with in vivo models found tumour regression and up to 66% increase in the 1-year survival when mice were treated with 1.9 nm Au NPs, compared to those non treated with NPs, under equal radiation conditions (Butterworth et al. 2010). Additionally, the damaging enhancement efficacy of NPs have been shown to improve when biodistribution is controlled by coating them with polyethylene glycol (PEG) (Geng et al. 2014; Liu et al. 2010; Zhang et al. 2012), and by conjugating them with targeting molecules as antibodies or other radiosensitizers (Wolfe et al. 2015).

Inorganic NPs can be also used in combination with near-infrared (NIR) photons (800–1100 nm) both for molecular imaging and selective photothermal therapy (Huang et al. 2006). Here, some Au NPs such as Au nanorods (Jana et al. 2001; Nikoobakht and El-Sayed 2003) or hollow Au NPs (Gonzalez et al. 2011) present a suitable strong surface plasmon resonance absorption in the NIR. This is a region of the light spectrum where there is a window of transparency for biological tissues (known as the therapeutic window) from the overlapping light absorption of water, haemoglobin, and melanin. Thus, the possibility to excite in the NIR region allows for both minimization photo damage of biological specimens and maximization the penetration depth into the tissue of the excitation light. Other examples include up-converting nanophosphors (UCNPs) (Haase and Schafer 2011), which exhibit photon upconversion: two or more incident photons within the NIR region are absorbed by the UCNPs and converted into one emitted photon with higher energy (Auzel 1973; Ostermayer 1971).

In addition, superparamagnetic NPs offer attractive possibilities to treat cancer by inducing hyperthermia (Giustini et al. 2010). Magnetic NPs can be manipulated by external magnetic fields which show intrinsic high penetrability into human tissue (McCarthy et al. 2007; Pankhurst et al. 2009). When these NPs are exposed to an alternating magnetic field of sufficient strength and frequency, there is a conversion of magnetic energy into thermal energy. The heat generated is then transferred to the cells surrounding the NPs, what can result in cancer cell death by apoptosis once the local temperature exceeds 40 °C and proteins denaturate (Pu et al. 2013; Wust et al. 2002). Several groups have shown a significant tumour inhibition during hyperthermia therapy by employing Fe_3_O_4_ NPs (Shinkai 2002). Although other heating technologies exist to perform hyperthermia (namely, optical heating using lasers and ultrasounds heating), the advantage of magnetic hyperthermia is that tumours located virtually anywhere in the human body can be treated (Petryk et al. 2013). Moreover, the same NPs can be used both for heating and magnetic resonance imaging (MRI) (Jiang et al. 2014).

**Fig. 2:**
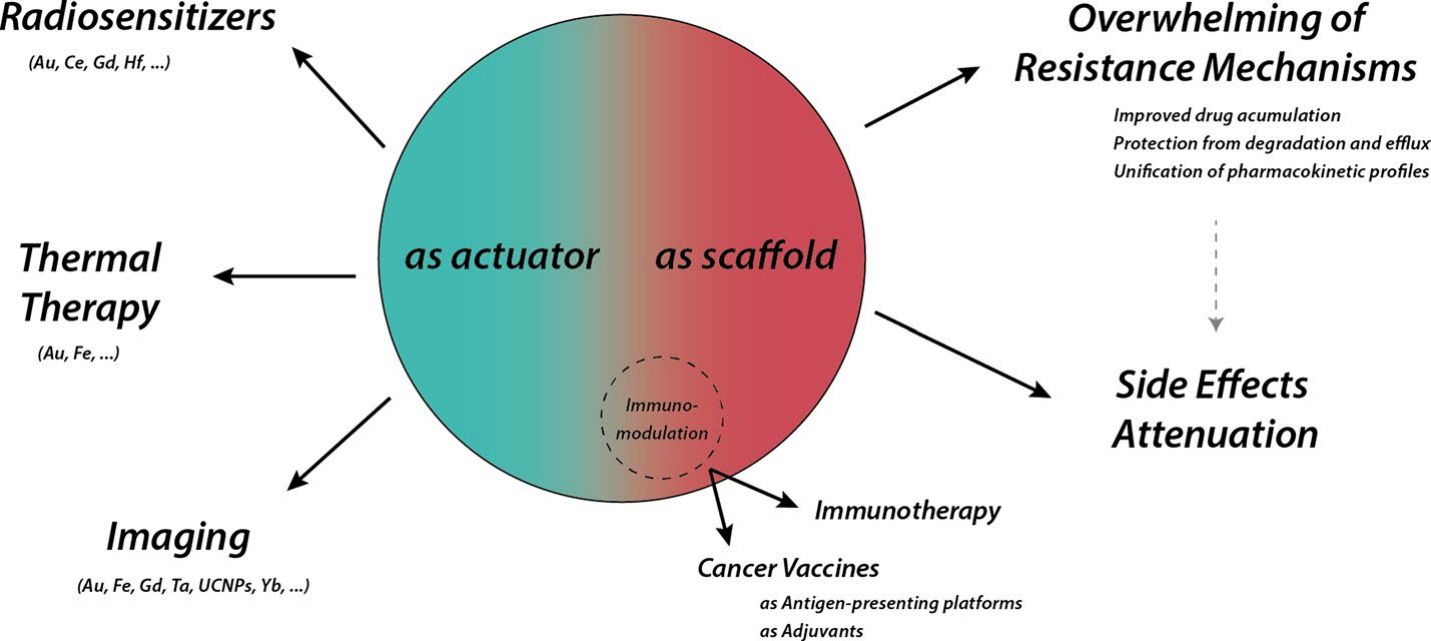
Different advantages enabled by a NP platform for a multimodal approach to address cancer resistance

An increasing body of literature supports the claim that the combination of different chemo, thermal, and radio treatment approaches significantly improves their outcome (Mi et al. 2012a). For instance, mild temperature hyperthermia has been proven to enhance sensitization to chemotherapy and radiotherapy through different mechanisms. First, local hyperthermia induces an increase of the blood flow and thus, greater concentrations of drugs can be delivered to tumours. Second, it also involves a higher oxygenation of the tissue, which further enhances the effect of radiotherapy (Song et al. 2005). On the other hand, the sequential use of chemotherapy and radiotherapy can also increase cancer cells radiosensitazion. The suggested molecular mechanism might rely on the effects of the chemotherapeutic drugs, dysregulating of S-phase checkpoints and inhibiting of the DNA-damage repair machinery, which potentiates the radiation-induced DNA damage (Lawrence et al. 2003). As a result, lower doses of radiation can be delivered and the side-effects to healthy organs are reduced (Fig. 2).

## Nanoparticles at work enabling multimodality

The combinatorial effects of the different therapies above mentioned may be the key for fighting resistance to treatment. Table 1 shows a summary of the defence mechanisms’ tumoural cells use to develop resistance, and the NP-based approach to simultaneously attack those mechanisms. In the literature of the past few years there are several examples of the combination of different therapies in a single NP platform. To our knowledge, the first studies showing promising results for multimodal therapy with NPs involved the co-delivery of biologically active molecules and drugs (*dual chemotherapy*). Thus, back in 2005, Sengupta et al. (2005) presented nanoparticulate system composed of a poly(lactic-*co*-glycolic acid) (PLGA)—doxorubicin-conjugated polymer core and a lipid shell consisting on PEG-distearoylphos-phatidylethanolamine, phosphatidylcholine and cholesterol. These NPs were subsequently loaded with combretastatin, a natural phenolic compound with capability to cause vascular disruption in tumours, thus combining chemotherapy and anti-angiogenesis therapy with encouraging results.

One year later, Wang et al. (2006) developed a variation of this previous NP with a hydrophobic cholesterol core, to uptake poorly water soluble drugs, and coated with a cationic polymer shell, to strongly attach to cell membranes, for the co-delivery of paclitaxel and a cytokine, the interleukin 12-encoded plasmid. The in vivo synergistic anticancer effect was demonstrated in a breast cancer model in mice. It showed that the tumour growth rate in mice treated with paclitaxel-loaded NP/IL-12-encoded plasmid complexes was significantly lower than that in the mice treated with either of the therapies alone (Wang et al. 2006). Apart from this, many other reports and reviews can be found easily in the literature highlighting the intense research efforts on this topic (Gao et al. 2015b; Kemp et al. 2016; Mi et al. 2012a; Shi et al. 2017; Zhang and Zhang 2016). Co-delivery of drugs has shown also better perfomance in overcoming cancer therapy resistance. For instance, the cocktail delivered with an acid-degradable core–shell NPs (MnSOD siRNA-delivering NPs made of a siRNA/poly(amidoamine) dendriplex core and an acid-degradable polyketal shell) was proved in reversing tamoxifen resistance (tamoxifen is an oestrogen receptor modulator agent that prevents oestrogen from binding to the oestrogen receptor and blocks breast cancer cell growth resistance in breast cancer (Cho et al. 2013) and rituximab-coated biodegradable polymer NPs loaded with both hydroxychloroquine and chlorambucil were proved to restore the sensitivity of chronic myelogenous leukaemia cells to cytotoxic targeted drugs (Mezzaroba et al. 2013), among many others.

Following to these ones, other studies started to focus on the *co* **-** *administration of chemotherapy, gene therapy and immune therapy* (Liu et al. 2011, 2014) agents using various carriers, which include inorganic NPs, peptides, liposomes, and polymeric NPs (Yuan et al. 2016). Regarding the use of inorganic NPs, recently, the study of Wu et al. (2017) used Buthionine sulfoxamide (BSO) to inhibit glutathione synthesis and celecoxib to down-regulte P-gp expression. Both molecules were co-loaded in polymer/inorganic hybrid NPs to form buthionine sulfoximine/celecoxib@biotin-heparin/heparin/calcium carbonate/calcium phosphate NPs (BSO/celecoxib@BNP). A reversal of MDR in the drug-resistant cells (MCF-7/ADR) pretreated by the dual-inhibitor loaded NPs was observed. Using other nanostructured systems, Gao et al. (2017) developed a pH-sensitive mixed micelles composed of HA and TPG copolymer to overcome MDR. These micelles increased intracellular uptake, (via CD44) receptor-mediated endocytosis, and further enhanced the drug accumulation in MCF-7/ADR cells and the reduction of the mitochondrial potential and ATP levels in cells. The copolymer micelles have been demonstrated to be a potential nanocarrier to overcome tumour MDR (Gao et al. 2017).

Similarly, many examples can be found on the advantages presented of the use inorganic NPs for *dual* **-** *modality therapy of cancer such as chemotherapy and photodynamic therapy.* For instance, Peng et al. (2009) used photosensitizing block copolymers and the SN-38 antitumoural drug in human colon cancer models. In the study of Liu et al. (2011) combination of chemotherapy and thermotherapy was carried out in docetaxel loaded PEGylated gold nanoshells on silica nanorattles for the ablation of hepatocellular carcinoma. Overcoming resistance with this dual (chemo and thermo) therapy has been also observed using trastuzumab-conjugated SiO_2_@AuNPs in trastuzumab-resistant breast cancer cells (Carpin et al. 2011), among many others (Yuan et al. 2016). More recently, Kievit et al. (2015) developed a NP-based siRNA delivery system comprised by a superparamagnetic iron oxide core (for magnetic hyperthermia) coated with chitosan, PEG, and PEI. This system knocked down Ape1 expression over 75% in medulloblastoma cells and ependymoma cells, and produced threefold greater sensitivity to ɣ-rays through synergetic effects.

Examples of combination of ionizing radiotherapy with other treatment modalities integrated in a single NP platform include, for instance, the study of Liu et al. (2015) that developed a core–shell nanostructure comprised by an upconversion NP core and mesoporous silica as the shell. The former acted as radiation dose amplifier, and the latter provides protection to the hypoxia-activated prodrug, tirapazamine (TPZ), which was loaded within the cavity between the core and shell. These NPs showed low cytotoxicity and high in vivo biocompatibility. As radiosensitizers, the TPZ-filled NPs exhibit a greatly enhanced cytotoxicity and anti-tumour efficacy, both in vivo and in vitro, compared with either free TPZ or RT alone. The group of Shi et al. (Fan et al. 2013) also reported a multifunctional up-conversion core/porous silica shell NPs loaded with cisplatin. In vitro and in vivo studies demonstrated an enhanced efficacy via synergetic chemo-/radiotherapy. Moreover, this system serves also as a diagnostic agent as it allowed simultaneous magnetic/luminiscent dual-mode imaging. An alternative approach to counteract radiation resistance is using siRNA to target related pathways. For instance, Nawroth et al. (2010) synthesized chitosan/siRNA NPs targeting TNFα and showed that administration of this complex completely prevented radiation-induced fibrosis in CDF1 mice, allowing for higher therapeutic doses.

Less examples are found for *triple* **-** *modality cancer treatment strategies.* Shi group further developed their up-conversion core/porous silica shell system by allowing the co-delivery of the radio-/photo-sensitizer haematoporphyrin (HP) and the radiosensitizer/chemodrug docetaxel (Fan et al. 2014). In vivo experiments showed the complete elimination of the tumour upon NIR and X-ray irradiation through synergetic chemo-/radio-/photodynamic therapy (Fan et al. 2014). Also, Mi et al. employed herceptin (immunotherapy) conjugated poly-lactic acid polymer NPs loaded with docetaxel (chemotherapy) and iron oxide NPs (thermotherapy) for the treatment of HER-2 positive breast cancer with encouraging results (Mi et al. 2012b).

Interestingly, the above-mentioned materials can be fused together in the same multimeric NP and thus Fe_3_O_4_ domains can be grown onto Au domains to combine magnetic and optical detection and excitation (Fantechi et al. 2017).

## The remaining challenge

As described in this review, nanoscale agents have been under intense research and exploited to enhance the delivery of drugs in the treatment of a number of diseases showing potential benefits in terms of pharmaceutical flexibility, selectivity, dose reduction, and minimization of adverse effects. Inorganic materials can also be used as imaging and radiotherapy agents demonstrating that NP-based therapies can act as “precision medicine” for targeting tumours and infections while leaving healthy tissue intact. However, despite the tremendous potential of nanomedicine and hundreds of millions (if not billions) poured from funding institutions, it could be acknowledged that little progress has been made towards matching expectations: while scientific community keep on trying new nanosized constructs in animal models looking for therapeutic efficacy, little progress is made towards proper knowledge of the processes involved, and if very promising results have been observed many times, it is irresponsible to imagine that it will be possible to master nanomedicine without a proper knowledge of the physical and chemical evolution of NPs inside living bodies. Recently, Derek Lowe’s comment on drug discovery and the pharma industry in the Science Magazine Blog (Lowe 2016), commenting on the nature materials paper analysis of NPs delivery to tumours (Wilhelm et al. 2016), recognized “Working out that delivery and pharmacokinetics aspects of these NPs was already known to be a challenge, but it’s proven to be even more of one than anybody thought” (Lowe 2016). Therefore, the following aspects of nanopharmacokinetics: what does the body to the NP rather than what does the NP to the body, and the consequences that this entails for the body and the NP are a key enabling-knowledge. Thus, the understanding of the precise evolution of imaging, irradiating, and delivery nanoplatforms inside the human body is a pressing need sine qua non to develop nanomedicine. Otherwise, we may face another decade of witchcraft where marvellous things with NPs are observed in the lab but never translated into the clinic to improve patient quality and expectation of life.

Thus, to enable the use of NPs in medicine, nanopharmacokinetics (ADME studies but adapted to NP characteristics) is needed. NPs evolve as they enter inside the body and body compartments, where the final working units are different from what was initially designed and produced. Consequently, it is necessary to understand the physico-chemical transformations and evolution of NPs inside biological systems in order to enable NPs to work precisely for medicine, understanding the mechanisms of action. It is being accepted that NPs may be destabilized when travelling through different parts of the body (Casals et al. 2008). Their high surface energy tend to aggregate them homogeneously (forming polycrystalline particles) or heterogeneously (with molecules and structures of the surroundings), both altering and modifying biodistribution. Similarly, during their time inside the body, the presence of different redox states (from rather reducing to clearly oxidizing), pH (the late endosome can go down to 5) and the presence of nucleophilic species and ionic scavengers, alter NP valence state and promote NP dissolution, especially in the small size range (Auffan et al. 2009). Inside the body, the protein absorption onto NP surface may not only modify NP surface properties but also result into protein changes (Goy-Lopez et al. 2012) and could alter their metabolization. The consequences of this change in the protein conformation and metabolization in, for example, the immune response, are still rather unknown.

The proper knowledge of the NPs physico-chemical state at all times of its evolution inside living bodies comprises among others the colloidal stability, vicinity interactions, chemical transformations-as corrosion-, association with plasma proteins-protein corona (PC)-, interaction with components of the immune system, and all the traditional ADME studies (administration, distribution, metabolization, and excretion of drugs from the body) but adapted to the unique NP specificities. This knowledge will enable to effectively design, produce and monitor the biological work of NPs and it will finally unleash nanomedicine potential.

## Abbreviations

APCs: antigen-presenting cells
CSC: cancer stem cells
CTLs: cytotoxic T-lymphocytes
DC: dendritic cells
EGRF: epidermal growth factor receptor
HER2: human epidermal growth factor receptor 2
LEEs: (secondary) low-energy electrons
MDR: multidrug resistance
MDSCs: myeloid-derived suppressor cells
MRI: magnetic resonance imaging
NIR: near-infrared
NK: natural killers
NP: nanoparticle
PLGA-NPs: poly(d, l-lactic-co-glycolic) acid NPs
TGF-b: transforming growth factor beta
TPZ: tirapazamine
Tregs: T-regulatory cells
UCNPs: up-converting nanophosphors
